# Antenatal Corticosteroids for Women at Risk of Imminent Preterm Birth in 7 sub-Saharan African Countries: A Policy and Implementation Landscape Analysis

**DOI:** 10.9745/GHSP-D-18-00171

**Published:** 2018-12-27

**Authors:** Dawn Greensides, Judith Robb-McCord, Angeline Noriega, James A. Litch

**Affiliations:** a Independent consultant, Johannesburg, South Africa.; b Every Preemie—SCALE/Project Concern International, Washington, DC, USA.; c Independent consultant, Portland, OR, USA.; d Every Preemie—SCALE/Global Alliance to Prevent Prematurity and Stillbirth (GAPPS), Seattle, WA, USA.

## Abstract

**Background:**

Every year approximately 15 million babies are born prematurely and nearly 1 million die due to preterm birth complications. Evidence shows that antenatal corticosteroids (ACS) can be used to improve preterm birth outcomes in particular clinical settings. We conducted a policy and implementation landscape analysis of ACS use for women at risk of imminent preterm birth in 7 low-income countries.

**Methods:**

A study framework and situation analysis tool were developed based on the World Health Organization (WHO) recommendation for ACS use among women at risk of preterm birth. The study was conducted in the Democratic Republic of the Congo, Ethiopia, Malawi, Nigeria, Sierra Leone, Tanzania, and Uganda. Primary data were collected through key informant interviews. Secondary data were gathered from publicly available sources, a survey of health management information system indicators, and demographic data from the Every Preemie—SCALE country profiles for preterm and low birth weight prevention and care.

**Results:**

All 7 countries are using ACS for women at risk of imminent preterm birth. The majority of countries include language on ACS use in clinical protocols or standard treatment guidelines; however, none include language on accurately measuring gestational age. For 2 of the 5 countries with national standards for ACS use, the upper gestational age limit for ACS use exceeded the WHO recommendation of 34 weeks. There are gaps in national guidance on how to determine if a woman is at risk of imminent preterm birth. Few countries include guidance that indicates ACS is contraindicated in the presence of infection. The majority of countries reported that facilities providing ACS meet comprehensive emergency obstetric and newborn care standards, and all countries reported the availability of some form of special newborn care or neonatal intensive care units at facilities providing ACS.

**Conclusions:**

Countries recognize challenges to access to high-quality maternal and newborn care that fulfill clinical care preconditions required for safe and effective ACS use. Key informants recommended support for clinical guidelines and provider training on ACS use, inclusion of obstetric indications for dexamethasone and betamethasone in national essential medicine lists, collecting and using ACS-related data, and improving the quality of maternal and newborn care, including specialized newborn care.

## Abstract (precis)

Countries have put in place some elements necessary for safe and effective antenatal corticosteroid (ACS) use, but significant challenges remain including: ensuring accurate gestational age determination, establishing clear treatment guidelines, strengthening provider capacity, incorporating obstetric indications for ACS use in national essential medicines lists, and collecting and using ACS-related data in the HMIS. Most importantly, the quality of maternal and newborn care, including specialized newborn care, needs improvement to ensure a strong foundation for the safe and effective use of ACS.

Résumé en français à la fin de l'article.

## BACKGROUND

Each year approximately 15 million babies are born prematurely (before 37 weeks of gestational age) and nearly 1 million die due to complications of preterm birth.^1^ Prematurity is the leading cause of newborn deaths in the first 4 weeks of life and the leading cause of death among children under age 5 around the world.^2^ Preterm birth is also a prominent cause of disability and ill health later in life.

In addition to essential newborn care and other more specialized postnatal care interventions, there is a body of evidence to support the use of specific maternal health interventions to improve preterm birth outcomes. These include magnesium sulfate, antibiotics for preterm labor, tocolytics, and a reduction in elective, early cesarean deliveries. Use of antenatal corticosteroids (ACS) for fetal lung maturation in select pregnant women who are at risk of imminent preterm birth is also widely acknowledged as an effective, evidence-based intervention to improve preterm birth outcomes.

Use of antenatal corticosteroids for fetal lung maturation in select pregnant women at risk of imminent preterm birth is widely acknowledged as effective to improve preterm birth outcomes.

The timely use of ACS for the management of preterm labor—before 34 weeks of gestation in high-resource settings with neonatal intensive care unit (NICU) services and with high certainty of gestation age estimation—has been associated with a 34% reduction in respiratory distress syndrome, 46% reduction in intraventricular hemorrhage, 54% reduction in necrotizing enterocolitis, and, overall, a 31% reduction in newborn mortality.^3^^–^^5^ Additional benefits include reduced length of hospital stay, lower rate of intensive care admissions, and reduced cost of care.^3^^–^^5^

Cochrane reviews have reported no benefit, and the potential for harm to newborns, when ACS is administered after 34 weeks of gestation, and an increased rate of puerperal sepsis.^3^^–^^5^ A World Health Organization (WHO) survey of facilities in 29 countries published in 2014 reported that more than 25% of ACS use occurred at gestational ages at which benefit is controversial or harmful.^6^ The Antenatal Corticosteroid Trial, a multicenter trial of ACS for management of preterm labor from 24 to 36 weeks of gestation in 6 lower-middle-income countries, reported increased newborn mortality and increased serious maternal infections in the intervention group, compared with the control group that received traditional care in peripheral health care settings with limited technology and without newborn intensive care.^7^

Guidance on the minimum requirements of maternal and newborn clinical support for safe and effective ACS use has been limited. The *WHO Recommendations on Interventions to Improve Preterm Birth Outcomes* (2015) made a strong recommendation in support of ACS use for women at risk of imminent preterm birth from 24 weeks to 34 weeks of gestation when 5 conditions are met^8^:

1. Accurate gestational age assessment
2. Preterm birth is imminent (within 7 days)
3. No clinical evidence of maternal infection exists
4. Adequate childbirth care is available
5. Adequate preterm newborn care is available

Five specific conditions are required for the safe and effective use of ACS, as recommended by WHO.

ACS is 1 of 13 lifesaving commodities identified in 2012 by the United Nations Commission on Life-Saving Commodities (UNCoLSC) for maternal, newborn, and child health. Projections made in 2012 indicated that the lives of an estimated 6 million women and children could be saved by 2017, if countries invested in these commodities and promoted health systems strengthening for improved access to and use of these commodities.^9^

The Reproductive, Maternal, Neonatal and Child Health Trust Fund supported 8 countries to implement the UNCoLSC recommendations. These 8 countries were designated as the “Pathfinder” countries in 2013. The UNCoLSC engaged the Pathfinder countries and established expert technical reference teams to advance the commission's agenda. The technical reference teams created technical working groups, spanning the 13 commodities and 10 recommendations, in order to focus on specific aspects of their agendas. The technical reference teams and related technical working groups advanced the UNCoLSC's recommended actions for 3 to 4 years and drew to a close in June 2016.

A multi-country analysis of health system bottlenecks and potential solutions for coverage of ACS found that 9 or more of 11 countries (more than 75%) in Africa and Asia reported very major or significant bottlenecks for health information systems (11 countries), essential medical products and technologies (9 out of 11 countries), and health service delivery (9 out of 11 countries).^10^ This survey highlighted the need for more specific information on the current use of ACS in Africa and Asia.

Because of the positive evidence supporting ACS use, low-resource countries are moving forward with its implementation to prevent preterm birth complications, which have contributed dramatically to newborn and under-5 mortality. This article presents findings of a policy and implementation landscape analysis of ACS use for women at risk of imminent preterm birth in 7 of the 8 Pathfinder countries: Democratic Republic of the Congo (DRC), Ethiopia, Malawi, Nigeria, Sierra Leone, Tanzania, and Uganda (excluding Senegal). The UNCoLSC Newborn Health Technical Reference Team commissioned the analysis and Every Preemie—SCALE, a cooperative agreement funded by the United States Agency for International Development (USAID), implemented it.^11^

This landscape analysis is intended to direct the attention of national and local stakeholders to important issues related to the safe and effective use of ACS in low-resource settings. Identifying these crucial needs can be pivotal in influencing policy change and driving responsive intervention development and implementation. Although the analysis did not enable us to scrutinize the actual quality of implementation, it provided valuable information regarding the framework for implementation in these 7 countries.

## METHODS

The study team used a framework and situation analysis tool to focus on public-sector services in 7 countries for this landscape analysis. We included the following UNCoLSC Pathfinder countries in the study: the DRC, Ethiopia, Malawi, Nigeria, Sierra Leone, Tanzania, and Uganda. However, Senegal was omitted from the study due to a lack of response from in-country stakeholders.

### Data Collection

We used primary qualitative research methods to collect information about ACS use through key informant interviews. Secondary quantitative data were gathered from publicly available sources in the 7 countries, including national standard treatment guidelines, essential medicines lists, drug formularies, national strategies and plans, national road maps, programmatic reports, and intrapartum protocols. If any of these sources were not readily available, we reached out to in-country contacts to obtain them where possible. See Table 1 for examples of select documents reviewed in each country. Secondary data were further supplemented by the 2015 *Health Management Information System Maternal and Newborn Health Indicator Survey*,^12^ conducted in 23 of USAID's priority maternal and child health countries. Demographic data from the Every Preemie—SCALE country profiles for preterm and low birth weight prevention and care,^13^ published in 2015, were also included as a fourth component. Additional secondary data were obtained from the most recent global Countdown to 2015 reports^14^^,^^15^ and a WHO survey on behalf of the UNCoLSC.^16^ We summarized the relevant information on the use of ACS in each country and used it to validate information provided by the key informants. Data collection and verification occurred from February to June 2016.

**TABLE 1.** Examples of Select Documents Reviewed by Country

Country	National Document
DRC	Maternal, Newborn, and Child Standards of Health User Manual, April 2015 Integrated Maternal, Newborn, and Child Standards for Health, Volume 2: Obstetric Emergency Care, April 2012 Obstetric Care Training and Neonatal Emergency Facilitators Guide, May 2012
Ethiopia	FMOH STGs for Primary Hospitals, 2014 FMOH STGs for General Hospitals, 2014 FMOH STGs for Health Centers, 2010 FMOH Management Protocol on Selected Obstetrics Topics for Health Centers, 2014 FMOH Basic Emergency Obstetric and Newborn Care Training Manual, 2013
Malawi	Malawi STGs Incorporating Malawi Essential Medicines List, 2015 Malawi National Reproductive Health Service Delivery Guidelines, 2014–2019 Participants Manual in Integrated Maternal and Neonatal Care, 2015 Reproductive Health Unit Obstetric Management Protocols
Nigeria	Report of Expert Consensus Panel on the use of ACS, October 2014 MOH National Strategic Health Development Plan 2010–2015 “Saving One Million Lives” Accelerating improvements in Nigeria's Health Outcomes through a new approach to basic services delivery, 2012
Sierra Leone	Basic Package of Essential Health Services for Sierra Leone 2015–2020, 2015 Reproductive, Newborn and Child Health Strategy 2011–2015 Maternity Africa: Policies and Guidelines for Intrapartum Postnatal and Neonatal Care Essential Obstetric and Newborn Care: Practical Guide for Midwives, Doctors With Obstetrics Training and Health Care Personnel Who Deal With Obstetric Emergencies, 2015
Tanzania	Administration of Antenatal Corticosteroids in Pre-Term Labour, July 2015, Guidelines Standard Treatment Guidelines and Essential Medicines List, Fourth Edition, 2013 Health Sector Strategic Plan July 2015–June 2020
Uganda	MOH Uganda Guidelines, 2012 MOH Uganda Clinical Guidelines and Essential Medicines and Health Supplies List for Uganda, 2012, Addendum 2: RMNCH Lifesaving Commodities Essential Medicines and Health Supplies List for Uganda, 2012

Abbreviations: ACS, antenatal corticosteroids; DRC, Democratic Republic of the Congo; FMOH, Federal Ministry of Health; MOH, Ministry of Health; RMNCH, reproductive, maternal, newborn, and child health; STG, standard treatment guidelines.

Interview questions were based on the 5 WHO conditions for safe ACS use and included whether or not ACS is in use in each country and at what level of care, and the availability of clinical guidelines to determine if a woman is at risk of imminent preterm birth, the presence of maternal infection, gestational age parameters for ACS use, and how to establish accurate measures for gestational age during pregnancy. In the analysis we also looked at the availability of comprehensive emergency obstetric care services and special newborn care services, including the availability of NICUs.

The key informant questionnaire focused on national-level ACS policy and implementation and was derived from a framework that laid out the overall objectives of the landscape analysis and key research questions. The framework was shared with members of the ACS Technical Working Group (under the UNCoLSC Newborn Health Technical Reference Team) for their review and input. Key informants were queried on the strengths of implementation as well as existing challenges and barriers.

Knowledgeable local and global experts nominated key informants in each of the Pathfinder countries, providing a purposeful sample. At least 1 key informant for every country was a senior-level ministry of health representative. Additional informants, if available, came from organizations working closely with the ministry of health for the respective country. In 7 of the 8 Pathfinder countries, key informants participated in interviews. Despite several attempts to reach suggested key informants in Senegal, efforts were unsuccessful. Senegal was therefore omitted from the study.

We interviewed key informants, including at least 1 senior ministry of health official in each country.

The study team conducted interviews with 1 to 4 key informants in each of the 7 countries. Representatives from each country had relevant experience and information regarding the use of ACS for women at imminent risk of preterm labor and were able to provide valuable insights.

Whenever possible, we conducted key informant interviews by phone or in person during a 4-week period between April 6 and May 6, 2016. We used a structured questionnaire with 29 defined questions for the key informant interviews. The questions promoted discussion and allowed for follow-up and clarification by the interviewer. Each interview took approximately 45 minutes. Three participants received an electronic copy of the written questionnaire to record their written responses due to challenges related to language or telephone connection issues. The questionnaire was also professionally translated into French for key informants from the DRC who preferred to provide written responses in French. See Table 2 for the number of key informants and method of interview by country.

**TABLE 2.** Number of Key Informants and Method of Interview by Country

Country	Number of Key Informants	Method of Interview
DRC	2	Phone interviews and written questionnaire
Ethiopia	1	Phone interview
Malawi	2	In-person interviews
Nigeria	1	Phone interview
Sierra Leone	4	Phone interviews and written questionnaire
Tanzania	1	Written questionnaire
Uganda	1	In-person interview

Abbreviation: DRC, Democratic Republic of the Congo.

### Data Analysis

In every case possible, we verified the key informant interview data using country-level documents obtained from the desk review. However, verification of key informant data was not possible for reported care practices or the quality of those practices, such as adequate childbirth care and preterm newborn care.

The study team made every effort to identify all of the available secondary information for review and analysis in each country. Although it is possible that we missed documents or did not identify a more up-to-date version, our team used multiple sources to identify the most current and relevant materials to mitigate this risk. In the case of inconsistencies between the raw data provided from the Countdown to 2015 reports,^14^^,^^15^ the WHO survey on behalf of the UNCoLSC, and the secondary data obtained through the desk review, the study team attempted to contact the authors of the Countdown to 2015 and WHO reports to obtain more information on the protocols used for their surveys to resolve inconsistencies.

## RESULTS

### ACS Use

ACS was approved for use at tertiary facilities in the DRC (based on information obtained from key informant interviews), Ethiopia,^20^ Malawi,^22^ Nigeria (key informant interview), Tanzania,^24^ and Uganda.^25^ A pre-referral first dose was also approved in Ethiopia, Tanzania, and Uganda before a patient transfers to a higher-level facility; however, key informant data indicated that ACS was not actually being implemented at lower-level facilities. See Table 3 for a list of countries and approved levels of care for ACS use, actual levels of care, and indications for use.

**TABLE 3.** ACS Use by Country: Level of Care, Indications for Use, and Pre-Referral Dose Authorization

Country	Level of Care Approved for ACS Use	Level of Care Where ACS Actually in Use	Indications for Use	Pre-Referral Dose Allowed
DRC	Tertiary and maternity hospitals	Tertiary and maternity hospital in capital only	pPROM, eclampsia, preterm labor	No
Ethiopia	Referral, general, primary hospitals, and health centers	Tertiary and secondary hospitals	Preterm labor	Yes
Malawi	Central and district hospitals	Central and district hospitals	Preterm labor	No
Nigeria	Tertiary hospitals	Tertiary hospitals	Preterm labor	No
Sierra Leone	Tertiary hospitals including district referral hospitals	Tertiary and district referral hospitals	None	No
Tanzania	Hospitals, health centers	Hospitals	Preterm labor	Yes
Uganda	Hospitals, health centers IV, III, and II	Hospitals, health center IV	pPROM and “risk of preterm delivery”	Yes

Abbreviations: ACS, antenatal corticosteroids; DRC, Democratic Republic of the Congo; pPROM, preterm premature rupture of the membranes.

Corticosteroids were on the national essential medicines lists for all countries, but they were not listed for obstetric indications in any of the 7 countries.

Corticosteroids were on the national essential medicines lists for all 7 countries, but not for obstetric indications.

### WHO Recommendations for the Safe and Effective Use of ACS

#### Policy, Clinical Protocols, and Guidelines

National-level policies and guidelines provide a critical foundation when adopting a new health care intervention. Each of the 7 countries had either a national ACS policy or guidelines, with indicated ACS use ranging from preterm labor alone to threatened preterm birth including severe preeclampsia/eclampsia, preterm premature rupture of membranes, and antepartum hemorrhage.

Based on our review of the most current clinical protocols and standard treatment guidelines in each of the 7 countries, there was variability among the countries on the inclusion of the 5 conditions for safe and effective use of ACS as stated in the *WHO Recommendations on Interventions to Improve Preterm Birth Outcomes*.^8^ See Table 4 for a summary of the 5 conditions and their inclusion in national clinical protocols or standard treatment guidelines by country.

**TABLE 4.** Inclusion of WHO Care Conditions Required for ACS Use in National Clinical Protocols or Standard Treatment Guidelines, by Country^a^

	WHO Condition for ACS Therapy				
	Gestational Age Can Be Accurately Undertaken	Preterm Birth Is Considered Imminent	No Clinical Evidence of Maternal Infection	Adequate Childbirth Care Is Available	Adequate Preterm Newborn Care Is Available
DRC	No	No	Yes	Yes	Yes
Ethiopia	No	Yes	Yes	Yes	Yes
Malawi	No	No	No	Yes	Yes
Nigeria	No	No	No	Yes	Yes
Sierra Leone	No info	No info	No info	No info	No info
Tanzania	No	No	No	Yes	No
Uganda	No	Yes	Yes	Yes	Yes

Abbreviations: ACS, antenatal corticosteroids; DRC, Democratic Republic of the Congo; WHO, World Health Organization.

^a^ Yes: WHO condition included in country protocols or guidelines; No: WHO condition not included in country protocols or guidelines.

#### Gestational Age Assessment

The national-level guidance on gestational age criteria for use of ACS varied between countries, ranging from 24 to 37 weeks, 28 to 34 weeks, and less than 34 weeks, with no minimum gestational age required. The DRC, Ethiopia, and Tanzania had national-level criteria for gestational ages that were appropriate for the safe use of ACS. Nigeria and Sierra Leone did not have a nationally standardized gestational age range, but key informants in Sierra Leone reported a commonly accepted gestational age range between 28 and 35 weeks. At the time of this writing, Nigeria was reportedly in the process of revising the standard treatment guidelines to include a gestational age of 30 to 34 weeks; however, current national clinical standards do not reflect this update. Importantly, Malawi and Uganda had national-level guidance for gestational age criteria that exceeded the 34-week upper limit. See Table 5 for gestational age parameters for ACS use and source by country.

**TABLE 5.** Gestational Age Parameters for ACS Use and Source by Country

Country	Gestational Age Criteria	Source
DRC	28–34 weeks	National-level document^19^
Ethiopia	28–34 weeks	National-level document^20^^,^^21^
Malawi	<34 weeks 28–34 weeks 24–37 weeks	National-level document^22^^,^^23^ (Note: Criteria varied between national documents available for review.)
Nigeria	30–34 weeks	Key informant, no national-level document
Sierra Leone	28–35 weeks	Key informant, no national-level document
Tanzania	28–34 weeks	National-level document^24^ (Note: ACS could be provided as low as 24 weeks if birth is at a well-equipped facility with a specialist available to manage the premature newborn.)
Uganda	32–37 weeks	National-level document^25^

Abbreviations: ACS, antenatal corticosteroids; DRC, Democratic Republic of the Congo.

National-level guidance on gestational age criteria varied between countries, from no minimum up to 37 weeks.

None of the 7 countries had national-level guidance on how to calculate gestational age relevant to preterm labor. In the Ethiopian *Management Protocol on Selected Obstetrics Topics for Health Centers*,^17^ there is information on how to determine gestational age, but it is found in the section on post-term pregnancy only.

#### Risk of Imminent Preterm Birth and Maternal Infection

Ethiopia was the only country to include language in its standard treatment guidelines on how to determine if a woman is at risk of imminent preterm birth. Only the clinical standards and guidelines in the DRC, Ethiopia, and Uganda indicated that ACS use is contraindicated in the presence of maternal infection. Nigeria was in the process of revising their standard treatment guidelines and reportedly planned to include how to assess gestational age and how to determine whether there is clinical evidence of infection, but the updated guidelines following this survey did not include this language.

#### Adequate Childbirth Care and Preterm Newborn Care

The majority of countries included language in their clinical protocols or standard treatment guidelines emphasizing the availability of adequate childbirth care. The DRC, Ethiopia, Malawi, Sierra Leone, Tanzania, and Uganda reported that facilities providing ACS met comprehensive emergency obstetric and newborn care standards. Nigeria stated that some, but not all, tertiary facilities providing ACS met comprehensive emergency obstetric and newborn care standards. Malawi reported that facilities authorized to provide pre-referral first-dose ACS at lower levels of the health care system met basic emergency obstetric and newborn care standards.

All countries reported that the preterm newborn care interventions recommended by WHO for safe and effective ACS use were available at facilities providing ACS, although they were not specifically stated as *required* to be in place to provide ACS. Key informant data also indicated that because these interventions were not required nor defined by the ministry of health, the availability, content, and quality of preterm newborn care interventions varied widely across facilities and countries. See Table 6 for preterm newborn care interventions that were reportedly available by country.

**TABLE 6.** Preterm Newborn Care Interventions Recommended by WHO for Safe and Effective ACS Use Reported by Key Informants as Being Available at Facilities Providing ACS, by Country

	Preterm Newborn Care Interventions				
Country	Resuscitation	Thermal Care	Infection Prevention and Treatment	Feeding Support	Safe Oxygen Use
DRC	Bag and mask	Skin-to-skin contact/KMC Incubators (not prevalent)	Handwashing Antibiotics Separate unit for sick babies	NG tube Daily weight monitoring Daily intake monitoring	Oxygen mixer Oxygen titration Pulse oximetry
Ethiopia	Bag and mask	Drying, cleaning, wrapping Skin-to-skin contact/KMC Radiant warmers/incubators	Handwashing Antibiotics Sterilization of equipment	Exclusive breastfeeding Expressed breast milk NG tube Daily weight monitoring	Oxygen titration Oxygen concentrator
Malawi	Bag and mask	Skin-to-skin contact Incubators (some available)	Handwashing Antibiotics Separate ward for sick babies	Expressed breast milk NG tube Daily weight monitoring	Oxygen mixer Oxygen titration Pulse oximetry
Nigeria	Bag and mask	KMC Incubators (at general hospitals)	Antibiotics Aseptic technique Separate ward for sick babies	Breastfeeding and all feeding alternatives NG tube (general and teaching hospitals)	Pulse oximetry
Sierra Leone	Bag and mask	Dry, warm, stimulate KMC	Antibiotics	Expressed breast milk Formula NG tube Daily weight monitoring	Oxygen mixer Pulse oximetry
Tanzania	Bag and mask	Skin-to-skin contact Incubators	Handwashing Antibiotics Separate ward for sick babies	Expressed breast milk NG tube Daily weight monitoring	Oxygen mixer Oxygen titration Pulse oximetry
Uganda	Bag and mask	Skin-to-skin contact Incubators (limited supply and irregular power supply)	Handwashing Antibiotics Separate ward for sick babies	Expressed breast milk NG tube Daily weight monitoring	Oxygen mixer/concentrator (limited use due to irregular power supply)

Abbreviations: ACS, antenatal corticosteroids; DRC, Democratic Republic of the Congo; NG, nasogastric; KMC, kangaroo mother care; WHO, World Health Organization.

All countries reported the availability of some form of special newborn care or the availability of NICUs. Wide variation existed among countries regarding the availability of NICUs where ACS is given. Only the DRC and Tanzania required a NICU to be in place in order to give ACS; however, Tanzania also reported that NICUs were often not available in facilities providing ACS. The DRC and Nigeria reported that NICUs were always present in facilities providing ACS, and Ethiopia, Malawi, and Uganda reported that NICUs were available in facilities that most often provided ACS. Sierra Leone's key informants reported that NICU care was available at 1 hospital only in Freetown and was limited in terms of quality of care.

Wide variation existed among countries regarding the availability of NICUs where ACS is given.

### ACS Prescription and Administration

In the DRC, Ethiopia, Nigeria, Tanzania, and Uganda, only high-level clinical practitioners such as doctors, including obstetrician-gynecologists, were nationally authorized to prescribe ACS. In addition to doctors, Malawi reportedly had medical or clinical officers prescribing ACS (key informant interview), and Ethiopia had a cadre of graduates from a masters in emergency surgery and obstetrics program who were also reportedly able to prescribe ACS (key informant interview). Nurses and midwives were allowed to administer ACS with clinical oversight but could not prescribe it in the DRC, Ethiopia, Malawi, Tanzania, and Uganda. In Ethiopia nurses and midwives at the health center level were also authorized to prescribe and administer a pre-referral first dose of ACS.

Sierra Leone did not have national guidance for ACS prescriptive authority or administration, but a key informant reported that doctors, including obstetrician-gynecologists, had prescriptive authority, and clinical health officers, medical officers, and midwives could administer ACS with clinical oversight.

### Clinical Training

Secondary data elaborating on the inclusion of ACS in health personnel preservice and in-service training were limited. Key informants in Ethiopia, Nigeria, and Uganda reported that ACS was included in their preservice clinical training materials. In addition, the DRC, Ethiopia, Malawi, Sierra Leone, Tanzania, and Uganda reported having ACS in their in-service clinical training materials. All countries listed training and capacity building for health care providers on ACS as an area of identified need for their programs. No information was collected on the content or comprehensiveness of either ACS preservice or in-service training materials or actual provider training.

### Health Metrics for ACS Use

None of the countries in the landscape analysis had an existing indicator for ACS use in their health management information system (HMIS). However, Ethiopia, Malawi, Nigeria, Tanzania, and Uganda had each proposed a national indicator for ACS to be integrated into their HMIS. Ethiopia, Malawi, and Tanzania had a nationally proposed indicator for ACS specifying “women less than 34 weeks receiving ACS.” Other proposed indicators included stock-out of ACS in the past month, hospitals providing ACS, women receiving steroids with delivery between 24 and 27 weeks of gestation, women receiving steroids with delivery between 28 and 34 weeks of gestation, and women in preterm labor receiving at least 1 dose of ACS before delivery.

None of the countries had an existing indicator for ACS use in their HMIS, but 5 of the countries had proposed one for future use.

Analysis of the 2015 *Health Management Information System Maternal and Newborn Health Indicator Survey* data revealed that 6 of the 7 countries (the DRC, Ethiopia, Malawi, Nigeria, Tanzania, and Uganda) captured data on a range of proxy indicators related to the 5 WHO preconditions for the safe and effective use of ACS.^12^ These included the number of antenatal care (ANC) visits (4 or more), maternal complications (preeclampsia/eclampsia) diagnosed in ANC, preterm birth as a complication diagnosed in labor and delivery, maternal complications diagnosed in labor and delivery (preterm premature rupture of membranes and antepartum hemorrhage), maternal gestational age measured in labor and delivery, maternal blood transfusion, essential newborn care including breastfeeding within 1 hour of birth and immediate skin-to-skin contact, and newborn resuscitation in labor and delivery.

All but 1 country captured data on proxy indicators related to 5 preconditions for the safe and effective use of ACS.

Each of the 7 countries was capturing the number of ANC visits and all but Nigeria captured gestational age (in weeks) in ANC. Malawi had an indicator for the diagnosis of preeclampsia/eclampsia in ANC, and Ethiopia, Malawi, and Tanzania had indicators for the diagnosis of antepartum hemorrhage in labor and delivery. The DRC was the only country with an indicator for active management of the third stage of labor, and both Malawi and Tanzania had indicators for cesarean delivery as a method of delivery. All countries but Nigeria had an indicator for breastfeeding within 1 hour of birth, and only Uganda had an indicator for immediate skin-to-skin contact as part of essential newborn care. Nigeria was the only country with an indicator for referral to Kangaroo Mother Care for postnatal care as part of managing newborn complications.

### Lessons Learned, Strengths, Opportunities, and Challenges

Key informants shared their country-specific views on lessons learned, strengths, opportunities, and challenges regarding the implementation of ACS in their countries (Table 7). Reported reasons for not implementing ACS at all levels of care where approved in the 7 Pathfinder countries included “inadequate newborn care at lower levels of care,” “ACS is not available,” “guidelines for ACS are not available at lower levels of care,” “staff at lower-level facilities are not adequately trained to provide ACS safely,” and “safety concerns due to outcomes of recent trials.”

**TABLE 7.** ACS Implementation Lessons Learned, Strengths, Opportunities, and Challenges Reported by Key Informants

**Lessons Learned**	ACS should be added to the EML for obstetric use Intervention needs to be cost-effective Need comprehensive package, not just guidelines Stakeholders need to be informed of new WHO recommendations Policies, guidelines, preservice education, in-service education, and regulatory bodies all need alignment Need increased community awareness of preterm birth
**Strengths**	Ministry of health involvement and ownership Guidelines and policies exist for most countries Strong political will and partner support exist for this intervention
**Opportunities**	Expand standards and guidelines to include assessment (i.e., how to determine who should receive ACS) Need studies of ACS impact at scale Partner commitment, political will Strengthen quality of ANC Increase community awareness to reduce delay in diagnosis
**Challenges**	Inadequate training and capacity building Inadequate information available to safely scale up the intervention in low-income countries Delay in diagnosis of preterm labor ACS not in preservice training Poor supply chain Lack of trained health care workers No specific implementation guidelines for new WHO recommendations Lack of diagnostics such as ultrasound to determine gestational age Many deliveries are occurring at lower-level facilities where it is not appropriate to provide ACS

Abbreviations: ACS, antenatal corticosteroids; ANC, antenatal care; EML, essential medicines list; WHO, World Health Organization.

## DISCUSSION

Nearly 2.2 million preterm births and approximately 195,000 direct preterm child deaths occur annually across the 7 Pathfinder countries highlighted in this analysis.^13^ Countries are responding to preterm birth as a public health priority and are moving to implement evidence-based interventions within national maternal and newborn health programs. ACS is one intervention for improved preterm birth outcomes. The safe and effective use of ACS, however, relies on the availability and quality of care across the continuum of care—from provision during preterm labor to childbirth and preterm newborn care—to improve newborn survival while limiting the risk of resultant maternal and newborn complications.

The *WHO Recommendations on Interventions to Improve Preterm Birth Outcomes*, based on a comprehensive review of evidence, provide necessary guidance for the safe and effective implementation of ACS for women at risk of imminent preterm birth from 24 weeks to 34 weeks of gestation when 5 conditions are met: (1) accurate gestational age assessment, (2) preterm birth is imminent (within 7 days), (3) no clinical evidence of maternal infection exists, (4) adequate childbirth care is available, and (5) adequate preterm newborn care is available.^8^ Further guidance on accurately estimating gestational age by WHO was made available in 2016. According to the *WHO Recommendations on Antenatal Care for a Positive Pregnancy Experience*, an ultrasound scan before 24 weeks of gestation (early ultrasound) is recommended for all pregnant women to accurately estimate gestational age.^18^

Each of the 7 countries included in this study was implementing ACS for women at risk of preterm birth and the majority of countries included language in their clinical protocols or standard treatment guidelines regarding the use of ACS. However, none of the countries included language on how to accurately assess gestational age, and there were gaps in national-level guidance or criteria for how to determine whether a woman is at risk of imminent preterm birth. The range of gestational age criteria for ACS use varied across countries, and only 3 countries aligned with the WHO recommendation of an upper limit of 34 weeks. At the same time, only a few countries included guidance indicating that ACS is contraindicated in the presence of infection. Existing standard treatment guidelines did, however, emphasize the need for adequate childbirth care and preterm newborn care when using ACS.

All national guidelines included language on ACS, but did not address how to assess gestational age or how to determine if a woman is at risk of imminent preterm birth.

ACS provision matched to the appropriate level of care facility is a major challenge for the safe and effective use of ACS. There was general agreement among the Pathfinder countries that ACS should be provided within a comprehensive package of maternal and newborn care that includes consistently available specialized (advanced) newborn care. Specific newborn care interventions listed as conditions for ACS use in the *WHO Recommendations on Interventions to Improve Preterm Birth Outcomes* are resuscitation, thermal care, infection prevention and treatment, feeding support, and safe oxygen use.^8^ The majority of countries reported that facilities providing ACS met comprehensive emergency obstetric and newborn care standards and all countries reported the availability of some form of special (advanced) newborn care or the availability of NICUs. However, countries reported that there was great variability in the quality and availability of these services across their health systems.

Most facilities providing ACS met comprehensive emergency obstetric and newborn care standards and all had some form of advanced newborn care or NICUs.

Access to health facility care with competent health care providers for maternal and newborn health in many low-income countries remains a persistent challenge and complicates the availability of the safe provision of ACS. According to the available population-based survey data, births at health facilities ranged from a low of 10% in Ethiopia to a high of 91% in Malawi, with an unweighted average of 31% across the 7 countries—leaving great room for improvement.^13^

HMIS indicators measuring the use of ACS were lacking in the Pathfinder countries, although most countries had proposed a national indicator for ACS use to be integrated into their HMIS. There were no suitable proxy indicators included in any of the countries' HMIS that are related to the 5 WHO preconditions for the safe and effective use of ACS. The 2015 *WHO Recommendations on Interventions to Improve Preterm Birth Outcomes*^8^ outlines these preconditions for use of ACS and should prompt the development of suitable proxy indicators.

Countries recognized that challenges remain in terms of consistent and high-quality maternal and newborn care that meet required clinical care preconditions to safely and effectively provide ACS. The evidence of benefit and harm associated with ACS use calls for caution to ensure that preconditions are met at service delivery points before providing ACS for threatened preterm birth. Each country identified specific areas to improve ACS implementation, for example, reestablishing gestational age guidelines to meet international recommendations, qualifying the type of providers and level of facility that can prescribe and administer ACS, the design and release of new standard treatment guidelines to direct ACS implementation, and developing e-learning curricula for ACS.

Key informants recommended continued support for several implementation components, including clinical guidelines for ACS use, inclusion of obstetric indications for dexamethasone and betamethasone in national essential medicines lists, strengthening the capacity of providers to safely provide ACS, measuring and collecting ACS-related data, and attention to the quality of childbirth care (comprehensive emergency obstetric and newborn care) and preterm newborn care. Key informants thought it would be useful to conduct in-depth facility-level quality of care surveys to more comprehensively evaluate the safe and effective implementation of maternal and newborn health services, including ACS use.

Emphasis was also placed on ways to address the need for accurate gestational age assessment, including making pregnancy test kits available in ANC to confirm pregnancy early before palpation is possible, ensuring the availability of ultrasound equipment in ANC, training ANC providers to use ultrasound technology for early assessment of fetal gestational age, and improving ANC provider competency to perform abdominal palpations and measure fundal height. Country representatives welcomed support for up-to-date technical briefs, training material, educational tools, and job aids.

Previous work on health system barriers to the uptake of ACS for preterm birth among 11 countries was implemented as part of the Every Newborn Action Plan process.^10^ The countries included in the ACS health system bottlenecks analysis that overlapped with countries in this landscape analysis are the DRC, Malawi, Nigeria, and Uganda. Findings highlighted in this landscape analysis that were also prioritized in the bottlenecks analysis include the need for clear guidelines on ACS use, the inclusion of ACS for fetal lung maturation on essential medicines lists, improved provider competency in the use of ACS, and defining indicators to track and monitor the use of ACS.

### Study Limitations

Due to the limited time and resources available for this study, we interviewed 1 to 4 key informants in each country. We recognize that this is a small number of key informants; however, study participants were not intended to be a representative sample of perspectives but rather to serve as key informants to provide essential information on current national policy, including supporting government documents. The key informants were nominated by knowledgeable local and global content experts, providing a purposeful sample. Interviews may not be representative of all views, or even the dominant view.

In every case possible, we verified the key informant interview data using country-level documentation obtained in the desk review. Verification of key informant data was not possible for care practices or the quality of those practices, such as adequate preterm newborn care and childbirth care. For Sierra Leone, the national-level standard treatment guidelines and the updated essential medicines list were unavailable.

This landscape analysis focuses on public-sector services and does not reflect the implementation of ACS in private-sector health facilities in the 7 countries.

Inconsistencies existed between the raw data provided from the Countdown to 2015 reports,^14^^,^^15^ the WHO survey on behalf of the UNCoLSC,^16^ and the secondary data obtained through the desk review. The research team attempted to gain information on how the raw data were gathered and validated but was unable to gain clarification on the protocols used by the Countdown and WHO/UNCoLSC surveys.

## CONCLUSIONS

The goal for ACS as an intervention within national maternal and newborn health programs is to improve health outcomes related to preterm birth. There are benefits and risks to the use of ACS, and implementation in low-resource settings must ensure consistently available, high-quality maternal and newborn health services and interventions that are safe and effective and adhere to the principle of “first do no harm.” This analysis has identified crucial needs for the safe and effective use of ACS at service delivery points that must be addressed by national and local stakeholders, for example, providing guidelines and means for accurately assessing gestational age and guidance on how to determine if a woman is at risk of imminent preterm birth. Ideally, the information provided in this analysis and ensuing conversations will meaningfully add to the global and national exchange regarding the safe and effective expansion of ACS and, ultimately, inform comprehensive programming for improved preterm birth outcomes.

## Notes

**En français**

**Corticostéroïdes anténatals chez les femmes présentant un risque de naissance prématurée imminente dans sept pays d'Afrique subsaharienne : Analyse des politiques et de leur mise en œuvre en contexte**

Les pays ont mis en place certains éléments nécessaires à une utilisation efficace et sûre des corticostéroïdes anténatals (ACS), mais de nombreux défis demeurent, notamment : la garantie de la détermination de l'âge gestationnel, l'élaboration de directives claires en matière de traitement, le renforcement des capacités des prestataires, l'inclusion d'indications obstétriques pour l'utilisation des ACS dans les listes nationales de médicaments essentiels, ainsi que la collecte et l'utilisation de données liées aux ACS dans les systèmes de gestion des informations de santé (HMIS). Plus important encore, la qualité des soins prodigués aux mères et aux nouveau-nés, y compris les soins néonatals spécialisés, doit être améliorée afin de disposer d'une assise solide pour l'utilisation efficace et sûre des ACS.

**RÉSUMÉ**

**Contexte :** Quinze millions de bébés environ naissent prématurément chaque année et près d'un million meurent à la suite de complications liées à une naissance prématurée. Il a été établi que les corticostéroïdes anténatals (ACS) peuvent être utilisés pour améliorer les issues associés à la naissance prématurée dans certaines conditions cliniques. Nous avons effectué une analyse des politiques et de leur mise en œuvre en contexte concernant l'utilisation des ACS chez les femmes présentant un risque de naissance prématurée dans sept pays à faible revenu.

**Méthodes :** Le cadre de l'étude et l'outil d'analyse de la situation ont été élaborées en se fondant sur les recommandations de l'Organisation mondiale de la Santé (OMS) relatives à l'utilisation des ACS chez les femmes présentant un risque de naissance prématurée. L'étude a été réalisée en République démocratique du Congo, en Éthiopie, au Malawi, au Nigeria, en Sierra-Leone, en Tanzanie et en Ouganda. Les données primaires ont été collectées au cours d'entretiens réalisés auprès d'informateurs clés. Les données secondaires ont été recueillies en se fondant sur les sources disponibles publiquement, une étude des indicateurs des systèmes de gestion des informations de santé et des données démographiques provenant des profils Every Preemie-SCALE des différents pays relatives aux soins des bébés présentant une insuffisance pondérale à la naissance et à la prévention de celle-ci.

**Résultats :** Les 7 pays ont recours aux ACS pour les femmes présentant un risque de naissance prématurée imminente. La majorité des pays inclut un libellé spécifique à l'utilisation des ACS dans les protocoles cliniques ou les directives thérapeutiques standard. Toutefois, aucun pays n'inclut de référence à la mesure précise de l'âge gestationnel. Dans deux des cinq pays disposant de normes nationales en matière d'utilisation des ACS, la limite d'âge gestationnel supérieur applicable à l'utilisation des ACS excédait la recommandation de l'OMS qui est de 34 semaines. Il existe des lacunes dans les directives nationales sur la manière de déterminer si une femme court un risque de naissance prématurée imminente. Peu de pays précisent dans leurs directives que les ACS sont contre-indiqués dans le cas d'une infection. La majorité des pays ont indiqué que les établissements de soins fournissant des ACS satisfont aux normes en matière de soins néonatals et obstétricaux d'urgence complets. De même, tous les pays ont signalé la présence d'unités de soins néonatals spéciaux ou de soins intensifs pour nouveau-nés dans les établissement dispensant les ACS.

**Conclusions :** Les pays reconnaissent les défis que posent l'accès à des soins maternels et néonatals de haute qualité qui remplissent les conditions préalables requises en termes de soins cliniques pour permettre une utilisation efficace et sûre des ACS. Certains informateurs clés ont recommandé l'élaboration de directives cliniques et la formation des prestataires de service à l'utilisation des ACS, l'inclusion des indications obstétriques du dexaméthasone et du bétaméthasones dans les listes nationales de médicaments essentiels, la collecte et l'utilisation des données liées aux ACS, ainsi que l'amélioration de la qualité des soins maternels et néonatals, y compris les soins spécialisés prodigués aux nouveau-nés.

## Notes

**Peer Reviewed**

**Funding:** The USAID Bureau for Global Health, Office of Maternal and Child Health and Nutrition and the UNCoLSC Newborn Health technical reference team provided funding for this landscape analysis.

**First Published Online:** December 20, 2018

© Greensides et al. This is an open-access article distributed under the terms of the Creative Commons Attribution 4.0 International License (CC BY 4.0), which permits unrestricted use, distribution, and reproduction in any medium, provided the original author and source are properly cited. To view a copy of the license, visit http://creativecommons.org/licenses/by/4.0/. When linking to this article, please use the following permanent link: https://doi.org/10.9745/GHSP-D-18-00171

## References

[1] Liu L Oza S Hogan D,. Global, regional, and national causes of child mortality in 2000–13, with projections to inform post-2015 priorities: an updated systematic analysis. Lancet. 2015;385(9966):430–440. 10.1016/S0140-6736(14)61698-6. 25280870

[2] Preterm birth. World Health Organization website. http://www.who.int/mediacentre/factsheets/fs363/en/. Published February 19, 2018. Accessed October 19, 2018.

[3] Roberts D Dalziel S. Antenatal corticosteroids for accelerating fetal lung maturation for women at risk of preterm birth. Cochrane Database Syst Rev. 2006;(3):CD004454. 10.1002/14651858.CD004454.pub2. 16856047

[4] Brownfoot FC Gagliardi DI Bain E Middleton P Crowther CA. Different corticosteroids and regimens for accelerating fetal lung maturation for women at risk of preterm birth. Cochrane Database Syst Rev. 2013;(8):CD006764. 10.1002/14651858.CD006764.pub3. 23990333

[5] Mwansa-Kambafwile J Cousens S Hansen T Lawn JE. Antenatal steroids in preterm labour for the prevention of neonatal deaths due to complications of preterm birth. Int J Epidemiol. 2010;39(Supplement 1):i122–i133. 10.1093/ije/dyq029. 20348115 PMC2845868

[6] Vogel JP Souza JP Gülmezoglu AM,; WHO Multi-Country Survey on Maternal and Newborn Health Research Network. Use of antenatal corticosteroids and tocolytic drugs in preterm births in 29 countries: an analysis of the WHO Multicountry Survey on Maternal and Newborn Health. Lancet. 2014;384(9957):1869–1877. 10.1016/S0140-6736(14)60580-8. 25128271

[7] Althabe F Belizán JM McClure EM,. A population-based, multifaceted strategy to implement antenatal corticosteroid treatment versus standard care for the reduction of neonatal mortality due to preterm birth in low-income and middle-income countries: the ACT cluster-randomised trial. Lancet. 2015;385(9968):629–639. 10.1016/S0140-6736(14)61651-2. 25458726 PMC4420619

[8] World Health Organization (WHO). WHO Recommendations on Interventions to Improve Preterm Birth Outcomes. Geneva: WHO; 2015. http://www.who.int/reproductivehealth/publications/maternal_perinatal_health/preterm-birth-guideline/en/. Accessed October 19, 2018. 26447264

[9] Every Woman Every Child. UN Commission on Life-Saving Commodities for Women and Children: Commissioner's Report. New York: Every Woman Every Child; 2012. https://www.unfpa.org/publications/un-commission-life-saving-commodities-women-and-children. Accessed October 19, 2018.

[10] Liu G Segrè J Gülmezoglu AM,; Working Group for UN Commission of Life Saving Commodities Antenatal Corticosteroids. Antenatal corticosteroids for management of preterm birth: a multi-country analysis of health system bottlenecks and potential solutions. BMC Pregnancy Childbirth. 2015;15(suppl 2):S3. 10.1186/1471-2393-15-S2-S3. 26390927 PMC4577756

[11] Every Preemie–SCALE website. http://www.everypreemie.org. Accessed October 19, 2018.

[12] Maternal and Child Survival Program (MCSP). Health Management Information System Maternal and Newborn Health Indicator Survey. Washington, DC: MCSP; 2015.

[13] Country Profiles. Every Preemie–SCALE website. http://www.everypreemie.org/country-profiles/. Accessed April 4, 2016.

[14] UNICEF; World Health Organization (WHO). A Decade of Tracking Progress for Maternal, Newborn and Child Survival: The 2015 Report. Geneva: WHO; 2015. http://countdown2030.org/documents/2015Report/Countdown_to_2015_final_report.pdf. Accessed October 19, 2018.

[15] UNICEF; World Health Organization (WHO). Fulfilling the Health Agenda for Women and Children: The 2014 Report. Geneva: WHO; 2014. http://countdown2030.org/2014-report. Accessed October 19, 2018.

[16] World Health Organization (WHO). Regulation and procurement of life-saving commodities for women and children in Every Woman Every Child (EWEC) countries 2015. Geneva: WHO; 2015.

[17] Federal Ministry of Health of Ethiopia. Management Protocol on Selected Obstetrics Topics for Health Centers. Ethiopia: Federal Ministry of Health; 2014.

[18] World Health Organization (WHO). WHO Recommendations on Antenatal Care for a Positive Pregnancy Experience. Geneva: WHO; 2016. https://www.who.int/reproductivehealth/publications/maternal_perinatal_health/anc-positive-pregnancy-experience/en/. Accessed October 19, 2018. 28079998

[19] Republique Democratique du Congo, Ministere de la Sante Publique. Normes de la zone de sante relatives aux interventions integrees de sante de la mere, du nouveau-ne et de l'enfant en Republique Democratique du Congo: Volume 2 Soins obstetricaux d'urgence. Republique Democratique du Congo: Ministere de la Sante Publique; 2012.

[20] Food, Medicine and Healthcare Administration and Control Authority of Ethiopia. Standard Treatment Guidelines for Primary Hospital. 3rd ed. Addis Ababa, Ethiopia: Food, Medicine and Healthcare Administration and Control Authority of Ethiopia; 2014. http://apps.who.int/medicinedocs/en/d/Js21693en/. Accessed October 19, 2018.

[21] Food, Medicine and Healthcare Administration and Control Authority of Ethiopia. Standard Treatment Guidelines for General Hospital. 3rd ed. Addis Ababa, Ethiopia: Food, Medicine and Healthcare Administration and Control Authority of Ethiopia; 2014. http://apps.who.int/medicinedocs/documents/s21694en/s21694en.pdf. Accessed October 19, 2018.

[22] Government of Malawi, Ministry of Health. Malawi Standard Treatment Guidelines (MSTG): Incorporating Malawi Essential Medicines List (MEML) 2015. 5th ed. Lilongwe, Malawi: Ministry of Health; 2015. http://apps.who.int/medicinedocs/documents/s23103en/s23103en.pdf. Accessed October 19, 2018.

[23] Government of Malawi, Ministry of Health. Every Newborn Action Plan: An Action Plan to End Preventable Neonatal Deaths in Malawi. Lilongwe, Malawi: Ministry of Health; 2015. http://www.who.int/pmnch/media/events/2015/malawi_enap.pdf. Accessed October 19, 2018.

[24] The United Republic of Tanzania, Ministry of Health and Social Welfare. Administration of Antenatal Corticosteroids in Pre-Term Labour. Dar es Salaam, Tanzania: Ministry of Health and Social Welfare; 2015.

[25] Republic of Uganda, Ministry of Health. Uganda Clinical Guidelines 2012: National Guidelines for Management of Common Conditions. Kampala, Uganda: Ministry of Health; 2012. http://apps.who.int/medicinedocs/en/d/Js21741en/. Accessed October 19, 2018.

